# Metaplastic breast cancers frequently express immune checkpoint markers FOXP3 and PD-L1

**DOI:** 10.1038/s41416-020-01065-3

**Published:** 2020-09-17

**Authors:** Emarene Kalaw, Malcolm Lim, Jamie R. Kutasovic, Anna Sokolova, Lucinda Taege, Kate Johnstone, James Bennett, Jodi M. Saunus, Colleen Niland, Kaltin Ferguson, Irma Gresshoff, Mark Bettington, Nirmala Pathmanathan, Gary M. Tse, David Papadimos, Rajadurai Pathmanathan, Gavin Harris, Rin Yamaguchi, Puay Hoon Tan, Stephen Fox, Sandra A. O’Toole, Peter T. Simpson, Sunil R. Lakhani, Amy E. McCart Reed

**Affiliations:** 1 Faculty of Medicine, UQ Centre for Clinical Research, The University of Queensland, Herston, Brisbane, QLD Australia; 2 QIMR Berghofer Medical Research Institute, Herston, Brisbane, QLD Australia; 3 Pathology Queensland, The Royal Brisbane & Women’s Hospital, Herston, Brisbane, QLD Australia; 4 Westmead Breast Cancer Institute, University of Sydney, Westmead, NSW Australia; 5 Department of Anatomical and Cellular Pathology, Prince of Wales Hospital, The Chinese University of Hong Kong, Sha Tin, Hong Kong; 6 Department of Histopathology, Sullivan Nicolaides Pathology, Bowen Hills, QLD Australia; 7 Jeffrey Cheah School of Medicine & Health Sciences, Monash University Malaysia, 47500 Bandar Sunway, Selangor Malaysia; 8 Canterbury Health Laboratories, Christchurch, New Zealand/Department of Molecular Medicine and Pathology, University of Auckland, Auckland, New Zealand; 9 Department of Pathology and Laboratory Medicine, Kurume University Medical Center, 155-1 Kokubu, Kurume‐shi, 839‐0863 Japan; 10 Division of Pathology, Singapore General Hospital, Singapore, Singapore; 11 Peter MacCallum Cancer Centre and University of Melbourne, Melbourne, VIC Australia; 12 Garvan Institute of Medical Research and the Kinghorn Cancer Centre, 384 Victoria Street, Darlinghurst, NSW 2010 Australia; 13 Department of Tissue Pathology and Diagnostic Oncology, Royal Prince Alfred Hospital, Camperdown, NSW 2050 Australia

**Keywords:** Breast cancer, Breast cancer, Tumour biomarkers

## Abstract

**Background:**

Metaplastic breast carcinoma encompasses a heterogeneous group of tumours with differentiation into squamous and/or spindle, chondroid, osseous or rhabdoid mesenchymal-looking elements. Emerging immunotherapies targeting Programmed Death Ligand 1 (PD-L1) and immune-suppressing T cells (Tregs) may benefit metaplastic breast cancer patients, which are typically chemo-resistant and do not express hormone therapy targets.

**Methods:**

We evaluated the immunohistochemical expression of PD-L1 and FOXP3, and the extent of tumour infiltrating lymphocytes (TILs) in a large cohort of metaplastic breast cancers, with survival data.

**Results:**

Metaplastic breast cancers were significantly enriched for PD-L1 positive tumour cells, compared to triple-negative ductal breast cancers (*P* < 0.0001), while there was no significant difference in PD-L1 positive TILs. Metaplastic breast cancers were also significantly enriched for TILs expressing FOXP3, with FOXP3 positive intra-tumoural TILs (iTILs) associated with an adverse prognostic outcome (*P* = 0.0226). Multivariate analysis identified FOXP3 iTILs expression status as an important independent prognostic factor for patient survival.

**Conclusions:**

Our findings indicate the clinical significance and prognostic value of FOXP3, PD-1/PD-L1 checkpoint and TILs in metaplastic breast cancer and confirm that a subset of metaplastics may benefit from immune-based therapies.

## Background

Metaplastic breast carcinoma (MBC) is a rare morphological subtype of breast cancer that accounts for 0.2–5% of invasive breast cancers.^1^ Inherently heterogeneous, MBC are characterised by neoplastic cells differentiating into heterologous elements including squamous, spindle, osseous, chondroid and others. MBC are classically triple-negative BC (TNBC), lacking expression of the clinically targetable biomarkers oestrogen- and progesterone-receptors (ER/PR) and HER2, leaving cytotoxic chemotherapy as the sole systemic therapeutic option. MBC have a poorer prognosis than matched TNBC of other morphological subtypes (non-MBC TNBC),^2–4^ and a poorer response to neo-adjuvant chemotherapy^5^. Overall, the TNBC clinical subtype of breast cancer is noted for its heterogeneity and can be stratified into four distinct molecular subtypes,^6^ with tumour infiltrating lymphocytes (TILs) likely to be higher in the basal-like and luminal androgen receptor subtypes;^7^ MBC are considered to be basal-like^8^ or, more recently, claudin-low.^9^

Tumour cells have the ability to directly suppress TILs or elicit an immunosuppressive response by recruitment and reactivation of immune subsets. TILs are a major prognostic indicator in TNBC^10,11^ and a universal scoring system for TILs is moving into diagnostic practice.^12^ PD-L1 (Programmed Death Ligand 1) expression on tumour cells can manipulate anti-tumour immune cells and dampen the immune response. In TNBC, PD-L1 expression is variably reported, with 20^13^–80%^14^ of samples positively stained, with no prognostic association;^14^ in an additional cohort, 30% of tumours were positive, and in this context, there was an association between PD-L1 expression with poor overall survival.^15^ Analyses of the KEYNOTE-086 study demonstrated durable responses to the anti-PD1 antibody, pembrolizumab, in a subset of pre-treated and untreated TNBC patients.^16,17^ In MBC, early reports show that PD-L1 was expressed in ~40% of cases (30/71 ref. ^18^, 2/5 ref. ^19^), but that this is unlikely to be driven by copy number alteration.^20^ Recently, a case report showed an extraordinary and durable response to anti-PD-1 therapy in combination with paclitaxel.^21^ Together, this furthers the prospect that immunotherapies may be useful in MBC. The role of regulatory T cells (Tregs) in immune suppression is clear, and much is being made of their potential for targeting in cancer therapy.^22^ FOXP3 is a key marker of Tregs, which exert immune suppressive functions. In TNBC, increases in FOXP3 positive Tregs are associated with improved survival,^23,24^ but also dismal overall survival in a meta-analysis of unselected breast cancers.^25^ Ultimately, little is known of FOXP3 expression in Tregs in MBC. In the current study, we assess TILs and the expression of PD-L1 and FOXP3 in the largest MBC cohort to date and explore the relationship between these features and patient outcome.

## Methods

### Clinical cohorts

Human research ethics committees approved the use of all clinical samples and data (The University of Queensland (2005000785) and the Royal Brisbane and Women’s Hospital (2005/022)). A subset of MBC cases from the Asia-Pacific Metaplastic Breast Cancer Consortium were assessed in this study,^26^ Table 1. The non-metaplastic TNBC cases from the Queensland Follow Up cohort were employed as a cross-sectional control cohort. Given the historical and multi-national nature of these cohorts, reliable treatment information is not available, however assumptions can be made, and high-grade cancers such as these would have indicated radiotherapy and chemotherapy as management strategies after surgery. Whole sections were analysed throughout the study.

**Table 1:** Metaplastic breast cancer cohort.

	*n*	%
Age		
<50	40	28.8
>51	99	71.2
Total	139	
Size		
<2 cm	31	21.4
2–5 cm	82	56.6
>5 cm	32	22.1
Total	145	
WHO Type		
1 (mixed)	116	71.6
2 (low‐grade adenosquamous)	1	0.6
3 (fibromatosis‐like)	4	2.5
4 (squamous)	25	15.4
5 (spindle)	14	8.6
6 (mesenchymal)	2	1.2
Total	162	
ER/PR/HER2		
TNBC	85	64.4
ER/PR pos	8	6.1
ER pos	13	9.8
PR pos	13	9.8
HER2 pos	13	9.8
Total	132	
TILs		
Grp 1: 0–10%	75	49.3
Grp 2: 20–40%	62	40.8
Grp 3: 50–90%	15	9.9
total	152	
PD-L1 tumour cells		
Positive	107	73
Negative	39	27
Total	146	
PD-L1 TILs		
Positive	91	63
Negative	53	37
Total	144	
FOXP3 sTILs		
Positive	73	49
Negative	76	51
Total	149	
FOXP3 iTILs		
Positive	57	38
Negative	93	62
Total	150	

### Pathology review

The morphological categorisation of the MBC was considered as per the WHO guidelines.^1,26^ MBC were classed as mixed (Type 1), squamous (Type 4) and spindle (Type 5), and the number of morphologies present in a mixed case were also noted. Stromal TILs were reported as an overall percentage of the stromal area (within the borders of the invasive tumour) covered by mononuclear immune cells. Tumours are classed as Group 1 (0–10%; immune cold); 2 (20–40%) or 3 (50–90%; immune hot). Care was taken to exclude any lymphocytic infiltrate around normal lobules, in the previous biopsy site or in areas of diathermy or crush artefact.^27^ We adhered to the requirements that: (1) evaluations be carried out within the borders of the invasive tumour and (2) TILs outside of border of the neoplasm and around DCIS be excluded.^12^ Two pathologists independently scored all criteria.

### Immunohistochemistry

For immunohistochemistry (IHC), heat-induced antigen retrieval was performed in pH 8.0 Tris-EDTA buffer at 95 °C for 30 min using NxGen Decloaking Chamber (Biocare Medical, Concord, CA, USA) followed by blocking with Background Sniper (Biocare Medical). FOXP3 (Clone D2W8E; Cell Signaling (Arundel, Queensland); 1:100) and PD-L1 (Clone E1L3N Cell Signaling; 1:200) were incubated for 2 h at room temperature. For β-catenin antibodies, antigen retrieval was in citrate buffer; Clone 14 (BD Biosciences, North Ryde, Australia), 1:200 dilution for a 1-hour incubation; Clone E247 (Abcam, Melbourne, Australia), 1:200 dilution incubated for 2 h. Antibodies were diluted in Da Vinci Green antibody diluent (Biocare Medical) and detected using MACH 1 Universal HRP-Polymer kit (Biocare Medical, Concord, CA, USA). Two pathologists independently scored all markers. A positive stain was defined as the presence of ≥5% of cells displaying unequivocal staining. PD-L1 tumour and TILs staining were assessed, while for FOXP3, stromal TILs (sTILs; no direct contact with malignant cells) and intratumoural TILs (iTILs; direct contact with malignant cells) were assessed^28^ as a count per high powered field (hpf). WNT signalling pathway activity was inferred from β-catenin staining,^29^ with nuclear and/or cytoplasmic staining suggestive of active signalling, and membrane-bound β-catenin indicating inactive WNT status). TILs staining was assessed only in those cases with 5% or more TILs. Histo-scores (staining intensity^1–3^ multiplied by percentage of cells stained) were calculated for each case.

### Statistical analysis

Data was analysed using Prism v8.2.0; tests as indicated, significance accepted as *P* < 0.05. Multivariate analysis was conducted using SPSS, with Forward Stepwise (Conditional LR) Method and a Regression Coefficient of 95.0% CI for Exp(B). The parameters used were tumour size, grade, LN positivity, Age, WHO types, TILs group, number of morphologies, lymphocytic infiltrate. PD-L1 tumour expression, PD-L1 sTILS expression, FOXP3 sTILS expression, and FOXP3 iTILS expression were tested as noted.

## Results

**Fig. 1:**
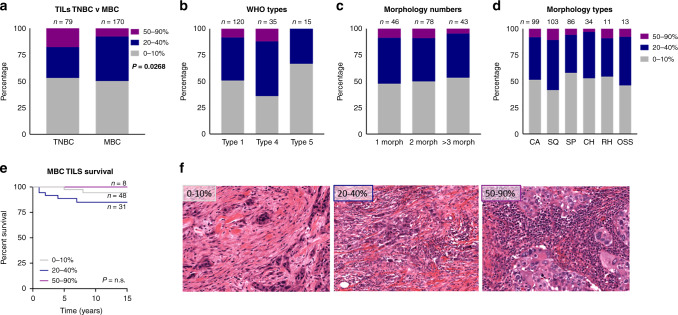
Tumour Infiltrating Lymphocytes in MBC. Contingency analysis of **a** TILs in MBC compared to unselected TNBC, with a Chi squared value of *P* = 0.0268; **b** WHO types 1 (mixed), 4 (squamous) and 5 (spindle), with no significant association; **c** numbers of morphologies (morph) present within the mixed (Type 1) MBC, with no significant association; and, **d** types of morphologies present within the mixed (Type 1) MBC (CA carcinoma; SQ squamous; SP spindle; CH chondroid; RH rhabdoid; OS osseous), with no significant association. **e** Kaplan–Meier survival curve assessing breast cancer-specific survival in MBCs, of the different proportions of TILs. **f** Representative examples of TILS distribution as determined by ref. ^12^ and in the MBC cohort; 0–10%; 20–40%; 50–90%).

Adhering to the International TILs Working group criteria,^12^ we assessed TILs across a cohort of metaplastic breast cancers (*n* = 170; Table 1). Almost half of the cohort was categorised immune cold (group 1; 49%), which is comparable to a cross-sectional TNBC cohort (53%, ref. ^30^). The MBCs, however, showed a significant reduction in the proportion of group 3 (immune hot) cases compared to TNBC (Fig. 1a; chi square *P* = 0.0268). There was no significant association between TILs and WHO Types, numbers of morphologies or morphology types (Fig. 1b, c, d. Chi square *P* = 0.35; *P* = 0.92; *P* = 0.72, respectively). TILs content did not significantly impact breast cancer-specific survival (BCSS) in the MBC cohort (Fig. 1e; Kaplan–Meier curve, Gehan-Breslow-Wilcoxon *P* = 0.11), representative images of TIL categories are shown in Fig. 1f.

**Fig. 2:**
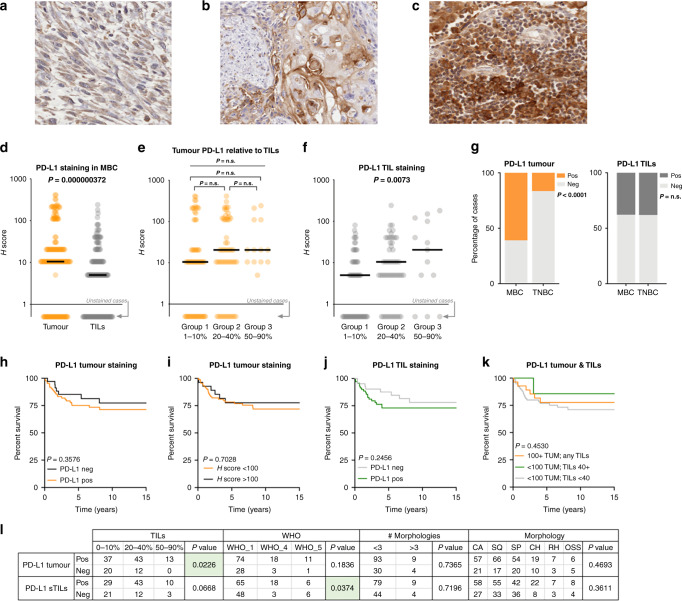
PD-L1 expression and prognostic implications in MBC. PD-L1 staining of tumour cells (**a**, **b**) and TILs (**c**). **d** Wilcoxon’s matched-pairs signed rank test (two tailed); note that cases with an absence of staining are indicated by the grey arrow. **e** Tumour PD-L1 expression relative to the level of TILs present, Kruskal–Wallis *P* = 0.2281 across the three groups; Mann–Whitney *U* tests were not significant between any of group 1 vs 2; 2 vs 3 and 1 vs 3 (*P* = 0.1957, 0.6698 and 0.1247, respectively). **f** Shows the TILs staining relative to TILs level, with a significant Kruskal–Wallis *P* = 0.0073. Note in the analyses of (**e**, **f**), group 1 (1–10%) includes only those cases with TILs. **g** Shows the proportion of cases containing PD-L1 expressing tumour cells (left) and TILs (right) in both the MBC and TNBC cohorts. Kaplan–Meier curves assessing breast cancer-specific survival are shown in **h** for PD-L1 tumour staining; **i** PD-L1 tumour staining with an H-score cut off of 100; **j** for TILs; and, **k** for a combination of tumour and TILs. **l** Contingency analyses (chi square test).

With increasing data to suggest that PD-L1 over-expression may be a useful marker for immunotherapy in MBC,^18,20,21^ we undertook a detailed characterisation of our large cohort. Predictably, PD-L1 staining patterns and histo-scores for both tumour and TILs staining across the diverse MBC morphologies was variable, and PD-L1 tumour staining presented as cytoplasmic or membranous (Fig. 2a, b). An example of strongly positive TILs staining is shown in Fig. 2c. A Wilcoxon’s matched-pairs signed rank test showed a strongly significant relationship between tumour (Fig. 2d) and TILs staining within a case, and with a higher average histo-score in the tumour. To determine the relationship between the level of TILs and PD-L1 staining, we stratified the cases into TILs groups (1, 1–10%; 2, 20–40%; 3, 50–90% infiltrate). Although average PD-L1 tumour staining appeared higher in TILs groups 2 and 3 it was not statistically significant (Fig. 2e). Intriguingly, there are cases classed as immune cold (TILs group 1) with high tumour PD-L1 staining (Fig. 2e). As expected, PD-L1 TILs staining increased with the number of TILs (*P* = 0.0073; Fig. 2f). However, comparing MBC to TNBC was striking, in that the MBC showed a significant increase in the tumour PD-L1 staining (Fig. 2g; *P* = 2.2 × 10^−11^), while no difference was observed for the TILs. No significant differences in BCSS were noted in relation to PD-L1 tumour staining (Fig. 2h, i), TILs staining (Fig. 2j), or a combination of tumour and TILs staining (Fig. 2k; with a tumour histo-score cut off of 100,^15^ and TILs of 40, which afforded the most stratification of the data).

Contingency analyses (Fig. 2l) identified significant relationships between PD-L1 tumour expression and increasing TIL presence (*P* = 0.0226) and PD-L1 positive TILs in WHO_4 (squamous) MBC type (*P* = 0.0374). In a multivariate assessment, PD-L1 tumour staining did not add any significance as a prognostic factor over lymph node (LN) positivity (*P* = 0.003; Table 2), and neither did PD-L1 TILs staining. We assessed TILs staining only in those cases with 5% or more TILs, and notably, three cases (7%) showed high tumoural PD-L1 expression in the absence of TILs.

**Table 2:** Multivariate analysis of PD-L1 and FOXP3 data.

			HR	95.0% CI	
Test	Variable	Sig.		Lower	Upper
PD-L1 tum	LN Pos	0.003	3.358	1.517	7.433
PD-L1 sTILs	LN Pos	0.005	3.167	1.412	7.101
FOXP3 sTILs	LN Pos	0.023	2.588	1.139	5.880
FOXP3 iTILs	LN Pos	0.027	2.547	1.114	5.824
	FOXP3 iTILs	0.042	2.372	1.033	5.448

^a^Forward step-wise conditional multivariate analysis containing the test (IHC data), LN positivity and grade.

*CI* confidence interval, *HR* hazard ratio, *Tum* tumour, *iTILs* tumour infiltrating lymphocytes, *Sig.* significance *P* value, *sTILs* stromal tumour infiltrating lymphocytes, *LN* lymph nodes.

**Fig. 3:**
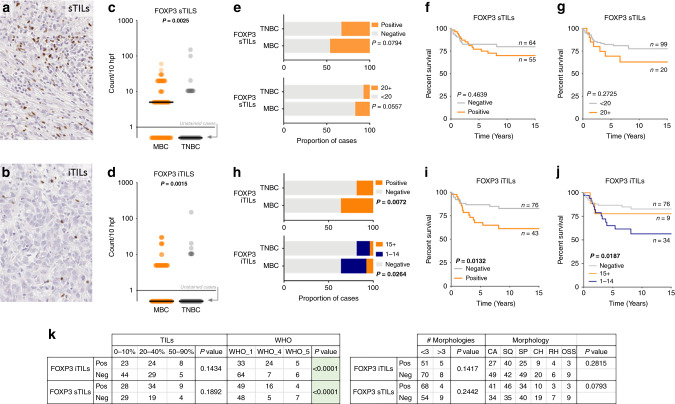
FOXP3 expression and prognostic implications in MBC. FOXP3 staining of sTILs (**a**) and iTILs (**b**). Wilcoxon’s matched-pairs signed rank test (two tailed) in **c**, sTILs (*P* = 0.0025) and **d** iTILs (*P* = 0.0015); note that cases with an absence of staining are indicated by the grey arrow. **e** Shows the proportion of cases containing FOXP3 expressing sTILs of the count cut off as shown in the survival analysis Kaplan–Meier curves assessing breast cancer-specific survival in **f**, **g** for sTILs with a positive/negative and <20 and 20+ count cut off, respectively. **h** Shows the proportion of cases containing FOXP3 expressing iTILs in both the MBC and TNBC cohorts, with cut offs of positive/negative and of <15 and 15+; Kaplan–Meier survival analysis for these groups is shown in **i**, **j** for iTILs. **k** Contingency analyses (chi square test).

**Fig. 4:**
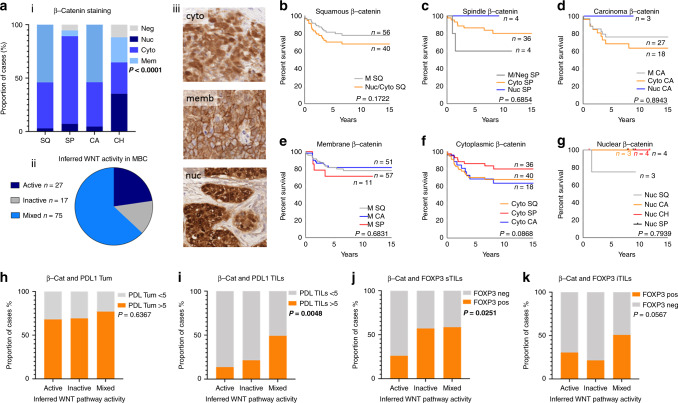
WNT activity and expression of immune checkpoint markers in MBC. WNT activity was determined using β-catenin staining as a surrogate. **a** (i) β-catenin sub-cellular localisation is significantly different across the phenotypic elements of MBC (Chi squared *P* < 0.0001); a pie chart (ii) shows most MBC cases assessed had mixed regions of active and inactive signalling; and, (iii) shows representative cytoplasmic, membrane and nuclear staining. Kaplan–Meier curves assessing breast cancer-specific survival are shown in **b** for tumours with squamous elements; **c** spindles; and, **d** carcinoma; **e** shows membranous staining; **f** shows cytoplasmic staining and, **g** shows nuclear staining. The relationship between WNT pathway activity and PD-L1 tumour expression (**h**) and TILs expression (**i**) as assessed by a Chi squared analysis (*P* = 0.6367 and *P* = 0.0048, respectively). The relationship between WNT pathway activity and FOXP3 stromal TILS expression (**j**) and iTILs expression (**k**) as assessed by a Chi squared analysis (*P* = 0.0251 and *P* = 0.0567, respectively). CA carcinoma; SQ squamous; SP spindle; CH chondroid; neg negative; nuc nuclear; memb membrane; cyto cytoplasmic.

We characterised the expression of FOXP3 expression in the sTILs (surrounding stroma) and the iTILs (intra-tumoural) of MBC, as has been previously defined as critical for the interpretation of FOXP3 staining^28^ (Fig. 3a, b) and found statistically significant differences in counts between MBC and TNBC, in both sTILs (Fig. 3c; *P* = 0.0025) and iTILs (Fig. 3d; *P* = 0.0015). Within the MBC cohort, 46% of cases show FOXP3+ sTILS and 36% of cases have iTILS that are FOXP3+ (Table 1 and Fig. 3e). While the TNBC show the greatest range of histo-scores, a median of zero and contingency analysis demonstrate only a slight, non-significant difference between TNBC and MBC (Fig. 3e; *P* = 0.0794). Presence or absence of FOXP3 staining in sTILs shows no appreciable impact on BCSS (Fig. 3f), however, a modified cut-off at a count/hpf of 20, displays a trend toward significance over time (Fig. 3g). Chi square analysis shows the increased number of FOXP3 + iTILs in MBC compared non-MBC TNBC (Fig. 3h); and FOXP3 + cases are associated with a significantly worse BCSS in MBC (Fig. 3i, j). Dissection of staining count cut-offs indicates there may be further complexities at play, with low level FOXP3 positivity conferring a poorer outcome than higher level and/or negative (Fig. 3j; *P* = 0.0187). Considering the metaplastic types and morphologies (Fig. 3k), cases lacking FOXP3 + iTILs were significantly enriched in the mixed metaplastics (WHO_1; *P* < 0.0001), while FOXP3 expressing iTILs and sTILs were enriched in the WHO_4 (squamous type; *P* < 0.0001), relative to the mixed and spindle subtypes. In a multivariate analysis, FOXP3 staining of sTILs added no further value than LN positivity, however FOXP3 iTILs were a significant prognostic indicator and were retained in the forward step-wise conditional model (*P* = 0.042; Table 2). Previous studies have indicated that tumoural PD-L1 expression is likely regulated by WNT signalling, especially in stem cells.^31^ We, therefore, used IHC assessment of β-catenin sub-cellular localisation as a surrogate marker of WNT activation^29^ to investigate the canonical ‘stem cell-like’ breast cancer, MBC. We identified a low overall activation level of WNT signalling (nuclear or cytoplasmic subcellular localisation β-catenin) across the MBC cohort with the β-catenin clone 14 antibody, with only 10 cases (of 120 informative cases) presenting nuclear staining, and an additional 17 cases with cytoplasmic staining. This was confirmed with the β-catenin clone E247 antibody (Abcam). Most commonly, we found membranous staining in squamous elements (indicating inactive WNT pathway status), frequently co-occurring with cytoplasmic staining; and, cytoplasmic staining in the spindle regions (Fig. 4a). Cytoplasmic expression of β-catenin conferred a trend towards a survival disadvantage to the squamous and carcinomatous element presenting MBC cases (Fig. 4b, d), while in spindle MBC (Fig. 4c, f) an active WNT (cytoplasmic/nuclear staining) conferred survival advantage. These detailed sub-cellular localisation analyses are limited by small numbers of cases. Membrane staining or WNT inactivity, did not differentiate survival outcomes between squamous and carcinoma patients, but showed a trend toward a poor outcome in spindle MBCs (Fig. 4e). Nuclear staining was present in insufficient numbers overall (Fig. 4g). We show that PD-L1 expression in the tumour is not significantly different when WNT is active (nuclear/cytoplasmic β-catenin expression) compared to inactive (membrane β-catenin staining) (Fig. 4h). In contrast, PD-L1 expression in TILs shows an inverse relationship, wherein there are increased numbers of cases with positive PD-L1 TILs that are considered WNT inactive (Fig. 4i). FOXP3 positive sTILS were more likely to be associated with an inactive/mixed WNT, whereas conversely, FOXP3 negative iTILs were more likely to be associated with an inactive WNT pathway (Fig. 4j, k) than mixed.

## Discussion

We present data demonstrating that MBC has a unique profile of expression of immune-regulatory markers PD-L1 and FOXP3, and that they differ significantly in their expression from the broader group of non-metaplastic TNBC. Indeed, we show that 73% of MBCs have tumoural expression of PD-L1, marking them as a suitable cohort for immunotherapy targeting the PD-1/PD-L1 checkpoint, especially in light of the impressive response to pembrolizumab as documented by Adams et al.^21^ We do not see any associations between PD-L1 or TILs, nor with BCSS in this MBC cohort. Intriguingly, the converse is true for FOXP3, with FOXP3 positive iTILs associating with a much poorer breast cancer-specific survival.

The PD-L1/PD-1 field is not without controversy, with conflicting guidelines for its application as a diagnostic marker. Our choice of antibody, E1L3N (Cell Signalling) was made following the detailed characterisation of PD-L1 in TNBC;^14^ that it is not implemented clinically is a limitation of this study. However, there are numerous inconsistencies between antibodies reported, and no clear guidelines for positivity determined, as elegantly discussed by Dill et al.^19^ For example, the Adams case report^21^ refers only to tumour cell expression in the account of durable response to pembrolizumab, while two recent assessments of the Phase 2 KEYNOTE-086 study of pembrolizumab in TNBC^16,17^ defined PD-L1 positivity as CPS ≥ 1, where CPS (combined positive score) is the ratio of PD-L1–positive cells (tumour cells, lymphocytes, and macrophages) to the total number of tumour cells × 100 (as validated by Kulungara et al.^32^). The Phase 1b KEYNOTE-012^33^ considered expression in stroma or ≥1% of tumour cells as PD-L1 positive. Ultimately, the FDA-approved SP142 companion diagnostic still courts controversy as the FDA-approved staining categories are not reproducible, sparking concern from world-renowned pathologists.^34^

This study has demonstrated that although MBC is more frequently immune-cold than TNBC, they have increased tumoural expression of PD-L1, and higher numbers of FOXP3 expressing TILs. A key finding of this study is that there exist patients with high tumour PD-L1 expression in the absence of abundant TILs, and this clinically relevant finding supports the need for checkpoint marker staining, rather than a solely morphology-based TILs assessment as a predictive biomarker. Equally, there exists data to support that patients with low or negative tumour PD-L1 expression may also benefit from PD-L1/PD-1 therapy (ref. ^35^ and references within). The research community awaits further data to confirm the framework for PD-L1/PD-1 predictive biomarkers.

Clinical development of strategies to target FOXP3 Tregs is underway^36^ with promising data on the reprogramming of Tregs through CD25 targeting emerging.^37^ Our data demonstrates that patients with FOXP3 positive iTILs have a poorer breast cancer-specific survival than those without FOXP3 positive iTILs, and as such, almost half of MBC patients may benefit from anti-FOXP3 therapies (55% of all MBC; a third of mixed MBC, three quarters of squamous MBC and half of spindle MBC). This contrasts with existing TNBC data,^23,24^ wherein FOXP3 positive TILs associated with an improved survival outcome but supports the work of Li et al., wherein patients with high PD-L1 and FOXP3 expression had poor survival^38^. A meta-analysis^25^ demonstrated that FOXP3 + sTILS and iTILs conferred a poorer prognosis in unselected breast cancer patients. Through Multivariate Cox regression we identified FOXP3 positive iTILs as an independent prognostic indicator in MBC.

Considering the relationship between PD-L1 and WNT signalling pathway activation, previous studies have indicated that PD-L1 is upregulated by WNT signalling in TNBC.^31^ Our data demonstrate that PD-L1 expression in the tumour is not significantly different when WNT is active (nuclear/cytoplasmic β-catenin expression) compared to inactive (membrane β-catenin staining). In contrast, PD-L1 expression in TILs shows an inverse relationship, wherein there are increased numbers of cases with positive PD-L1 TILs that are considered WNT inactive. Further studies are needed to tease out the links, if any, between PD-L1 expression and WNT signalling in metaplastic breast cancers.

In summary, we corroborate the case-study informed suggestion that the MBC cohort may benefit from PD-L1/PD-1 targeted immunotherapy, we show that Treg modulation may also be therapeutically important in this cohort and provide evidence that the immune-microenvironment of MBC does not mirror that of TNBC. Together, this provides the basis for further studies to open up a new avenue of therapeutic strategies for this other-wise under-served breast cancer population.

## Data Availability

All data is included in the publication.
